# Antibiotic utilization study in a teaching hospital in Nigeria

**DOI:** 10.1093/jacamr/dlac093

**Published:** 2022-09-05

**Authors:** Kehinde F Sekoni, Ibrahim A Oreagba, Farouk A Oladoja

**Affiliations:** Department of Pharmacology, Therapeutics and Toxicology Faculty of Basic Medical Science, University of Lagos, Akoka, Lagos-State, Nigeria; Department of Pharmacology, Therapeutics and Toxicology Faculty of Basic Medical Science, University of Lagos, Akoka, Lagos-State, Nigeria; Department of Pharmacology and Toxicology, Faculty of Pharmacy, Olabisi Onabanjo University, Ago-Iwoye, Ogun-State, Nigeria

## Abstract

**Background:**

Antibiotics have been the bedrock of modern medical care, particularly bacterial infections. However, globally, antimicrobial resistance has become a well-recognized public health threat in recent years, and interventions to reduce its burden have been launched worldwide.

**Objectives:**

The present study evaluated antibiotic utilization in both hospitalized patients and outpatients in a University Hospital in Nigeria.

**Methods:**

In a 3 year retrospective study between January 2017 and December 2019, 246 case files of patients were selected for the study based on inclusion and exclusion criteria. In addition, the antibiotic consumption rate for hospitalized and outpatients was determined.

**Results:**

The total antibiotic consumption for hospitalized patients in this study was 260.9 DDD/100 bed-days, while the outpatient department’s patients were 72.3 DDD/1000 inhabitants per day. Peptic ulcer disease was the most frequent indication for antibiotic use for outpatients, with the fluoroquinolones and macrolides being the most prescribed antibiotic class and antibiotic class with the highest DDD, respectively. The most frequent indication for antibiotic use for hospitalized patients was chronic kidney diseases, with the fluoroquinolones and second-generation cephalosporins being the most prescribed antibiotic class and antibiotic class with the highest DDD, respectively. DDD per 100 bed-days and DDD per 1000 patient-days were highest in 2018. The *P* values for the years were 0.019, 0.195 and 0.001 for 2017, 2018 and 2019, respectively.

**Conclusions:**

Our findings revealed irrationality in antibiotic use. Therefore, antimicrobial stewardship programmes should be implemented.

## Introduction

Antimicrobial resistance has recently been recognized as a significant determining factor for morbidity, mortality and increased cost in hospitals, with optimization of antibiotic utilization as one of the strategies for battling this scourge.^1^

The optimization of antibiotic utilization, at its most basic level, is the appropriate use of antibiotics and the limiting of unnecessary antibiotic administration/exposure, which consist of appropriate diagnosis, acquiring the appropriate culture and susceptibility data, implementing the most appropriate treatment, selecting the most effective antibiotics and dosing the antibiotics appropriately.^2^

In Nigeria, where there is a high level of poverty and poor healthcare financing, the majority of its citizens would only visit the hospital when the illness becomes life-threatening and would have employed all forms of quick solutions to relieve the burden of disease, which include inappropriate use of medicines that contributes to the growing antimicrobial resistance.^3^

Un-curtailed access to antibiotics; poor regulatory policies; poor infection control practices; lack of treatment guidelines or, where they exist, poor prescribers’ compliance; patient pressure; and pharmaceutical companies’ pressure on physicians are all major contributing factors to antimicrobial resistance.^4^

To combat the marked rise in antimicrobial resistance, WHO advocates the adoption of antimicrobial stewardship by healthcare providers to check and reduce the burden of antibiotic resistance.^5^ The strategy involves the application of objective interventions to influence prescribing practices, thereby promoting rational and appropriate antimicrobial use. The intervention is vital in developing countries, which usually have a combination of poor antimicrobial prescribing practices, unregulated over-the-counter sale of antibiotics and increasing rates of antimicrobial resistance.^6^

Due to the change in prescribing patterns and in the face of newer drug formulations and ever-emerging antibiotic resistance, it is pertinent that data be obtained on antibiotic utilization in different geopolitical zones of the country. In addition, accurate information about prescribing patterns in hospitals is valuable in improving the quality of antimicrobial utilization.^7^ However, most studies on antibiotic utilization focused on antibiotic use in specific departments or compared antibiotic utilization between hospitals.

It is necessary to carry out a retrospective study on how antibiotics are used in our tertiary hospitals using the ATC/DDD methodology as a basis of comparison and subsequently use the findings to develop and implement tailored strategies and interventions towards promoting appropriate use of antimicrobials in the future.

This study aims to evaluate the utilization of antibiotics in hospitalized patients and patients visiting the general outpatient department of Lagos University Teaching Hospital using a retrospective study.

## Methods

### Study design

A 3 year retrospective study from January 2017 to December 2019 focused on antibiotic utilization in the hospitalized patients and outpatients at Lagos University Teaching Hospital, Southwestern Nigeria.

This survey also includes descriptive variables of the ward- and patient-level data to describe the extent of antibiotic use in the hospital.

The method used for reviewing the medical records was primarily active search of presence of antibiotics in the records of patients meeting the inclusion criteria. Th system for medical recording used in the hospital was paper based. The records were reviewed by the authors K.F.S. and F.A.O. The process was verified by senior officials of the Health Records Department, Lagos University Teaching Hospital.

### Sample size

The reported prevalence of antibiotic use in both inpatient and outpatient settings is more than 80% in Kenya.^8^ The Z value of 1.96 and 5% level of significance was used to calculate the sample size using the formula below:

Where; p = prevalence; q = (1 − p); d = level of significance. Hence, *N* = 246 patients.

A total number of 246 case notes and medical records of patients who either visited the general outpatient department or were hospitalized and met the inclusion criteria were reviewed.

### Antibiotic inclusion criteria

Antibiotics which were classified as J01 category (antibiotics for systemic use) under the ATC classification system were included in this study. The number of antibiotics dispensed for 36 consecutive months was consulted, and data were extracted from medical records using a table with the antibiotic’s name, strength, amount dispensed and date dispensed.

### Inclusion criteria for the study

Adults admitted to the hospital and those visiting the outpatient department for both genders on antibiotic therapy in the medicine department were included.

### Exclusion criteria

Surgical patients, pregnant and nursing mothers, patients on chemotherapy, paediatric patients, emergency cases and short-stay cases were excluded.

### Study variables

Potential datasets for the study shall include the following: (i) details of the wards for data collection; and (ii) dose, frequency, route of administration and length of treatment of all antibiotics prescribed for each patient.

### Calculation of antibiotic consumption rate

The number of DDDs was calculated by first converting the total amount of antibiotics dispensed into grams divided by the standard WHO DDD value given in grams. For example, DDD/100 bed-days is given by dividing the number of DDDs by patient-days and multiplying by 100.

### Data analysis

Data analysis was undertaken using Microsoft Excel 2010 and statistical software SPSS version 23. The analysis of variance (ANOVA) was used for continuous variables as appropriate, while χ^2^ was used to evaluate categorical associations (*P* < 0.05 was considered significant).

### Ethical considerations

Ethical clearance to carry out the study was obtained from the Ethics Committee of the Lagos University Teaching hospital (CMUL HREC), Idi-araba, Lagos, Nigeria, with an assigned number: ADM/DCST/HREC/APP/3970.

## Results

### Demographic distribution and analysis of drug utilization pattern

Of the 246 patients selected, 60 were hospitalized patients and 186 were patients who visited the general outpatient department. For outpatients, 124 were female and 62 were male, with the IQR of antibiotics per prescription being 1–2 antibiotics.

For patients admitted, 35 were female and 25 were male. The median age for outpatients was 43 years, while that of hospitalized patients was 45 years. At least one person received a prescription of six antibiotics during the entirety of their stay. Eight people received only one antibiotic, 15 patients received two antibiotics, 11 patients received three antibiotics, 18 patients received four antibiotics and 7 received five antibiotics. The IQR of antibiotics per prescription for the hospitalized patients is in the range of 2–4 (Table 1).

**Table 1.** Demographic distribution and analysis of drug utilization pattern

Variable	Outpatient (*n* = 186)	Inpatient (*n* = 60)
Female, *n* (%)	124 (66.67)	35 (58.33)
Male, *n* (%)	62 (33.33)	25 (41.67)
Age, years, median (IQR)	43.00 (30.00–55.25)	45.00 (27.00-47.50)
Number of antibiotics per prescription, median (IQR)	1 (1.00–2.00)	3 (2.00–4.00)
Number of antibiotics per patient		
1	130 (69.89)	8 (13.33)
2	46 (24.73)	15 (25.00)
3	9 (4.84)	11 (18.33)
4	1 (0.54)	18 (30.00)
5	0 (0.00)	7 (11.67)
6	0 (0.00)	1 (1.67)

### Analysis of indication of antibiotic utilization for outpatients

Our findings revealed that of the 186 outpatients, 46, 28, 16 and 14 persons whose medical records were reviewed were treated for peptic ulcer disease, urinary tract infection, respiratory tract infection and bacterial vaginosis, respectively (Table 2).

**Table 2.** Analysis of indications for antibiotic use in general outpatient department

Ailment	*n*	Frequency
Abdominal pain	1	0.39
Appendicitis	2	0.78
Asthma	10	3.89
Avulsion of secondary trauma	1	0.39
Bacterial vaginosis	14	5.45
Bilateral epididymitis	1	0.39
Candidiasis	7	2.72
Cellulitis	6	2.33
Chest pain	1	0.39
Cholelithiasis	1	0.39
Chronic diarrhoea	3	1.17
Chronic liver disease	2	0.78
Chronic sinusitis	1	0.39
Chronic suppurative otitis media	1	0.39
Cyesis	1	0.39
Cystitis	1	0.39
Dactylitis	1	0.39
Dog bite with cellulitis	1	0.39
Dyspepsia	5	1.95
Enteritis	4	1.56
Epididymo-orchitis	1	0.39
Furunculosis	3	1.17
Gastritis	6	2.33
Gastroenteritis	10	3.89
Gastroesophageal reflux disease	2	0.78
Genital herpes	1	0.39
Gonorrhoea	3	1.17
Insomnia	1	0.39
Irritable bowel syndrome	3	1.17
Lobar Pneumonia	2	0.78
Malaria	5	1.95
MDR urinary tract infection	2	0.78
Nephrolithiasis	1	0.39
Osteoarthritis	1	0.39
Otitis	3	1.17
Pelvic inflammatory disease	14	5.45
Peptic ulcer disease	46	17.90
Pharyngotonsillitis	3	1.17
Phlebitis	1	0.39
Poor glycaemic control	2	0.78
Pyelonephritis	2	0.78
Respiratory tract infection	16	6.23
Retinopathy	2	0.78
Rhinitis	2	0.78
Rhinosinusitis	3	1.17
Right otitis externa	1	0.39
Right shoulder abscess	1	0.39
Sepsis	3	1.17
Septic shock secondary to Rt pyelonephritis	3	1.17
Sexually transmitted infection with oligospermia	5	1.95
Sexually transmitted infection	2	0.78
Smoke inhalation induced cough	1	0.39
Subacute appendicitis	3	1.17
Superficial burns	2	0.78
Temporal arteritis	1	0.39
Tonsilitis	4	1.56
Ulcer of right leg	1	0.39
Unilateral tonsilitis	1	0.39
Urethritis	3	1.17
Urinary tract infection	29	11.20
Uvulitis	1	0.39
Vaginal candidiasis	3	1.17
Wound	1	0.39

### Analysis of indication of antibiotic utilization for hospitalized patients

For hospitalized patients, findings revealed chronic kidney disease was the most frequent cause of hospitalization that warranted antibiotic use (*n* = 15), followed by bilateral vestibular failure (*n* = 13). Five persons were treated with antibiotics for back pain with cholelithiasis, malaria, gastroenteritis and gluteal ulcer, having the same number of hospitalized patients on antibiotics (*n* = 4) (Table 3).

**Table 3.** Distribution of indications of antibiotic use for hospitalized patients

Ailment	*n*	%
Aplastic anaemia with SIH	3	3.80
Back pain	5	6.33
Bell’s palsy	1	1.27
Bilateral pyelonephritis	3	3.80
Bilateral ventricular failure	13	16.46
Bloody urine	2	2.53
Brainstem tumour	2	2.53
Cholelithiasis	4	5.06
Chronic kidney disease	15	18.99
Community-acquired pneumonia	2	2.53
Congestive cardiac failure	2	2.53
Deep vein thrombosis	3	3.80
DMFS	1	1.27
Gastritis	2	2.53
Gastroenteritis	4	5.06
Gluteal ulcer	4	5.06
Haematuria	3	3.80
Hyperglycaemic crisis	1	1.27
Malaria	4	5.06
Meningitis	3	3.80
Nephrotic syndrome	2	2.53

### Analysis of antibiotic use

Findings revealed the majority of the patients (*n* = 46) received amoxicillin and clavulanic acid combination drugs, followed by ciprofloxacin (*n* = 34), amoxicillin (*n* = 28), clarithromycin (*n* = 26), metronidazole (*n* = 25), levofloxacin (*n* = 17), azithromycin (*n* = 15) and cefuroxime (*n* = 12) followed by the other antibiotics as shown in Table 4.

**Table 4.** Distribution of outpatient antibiotic use

Antibiotics	ATC	Route	*n*	%
Amikacin	J01GB01	Parenteral	1	0.39
Amoxicillin	J01CA04	Oral	28	10.85
Amoxicillin and clavulanic acid	J01CR02	Oral	46	17.83
Amoxicillin and flucloxacillin	J01CR0	Oral	4	1.55
Ampicillin and cloxacillin	J01CR50	Oral	2	0.78
Azithromycin	J01FA10	Oral	15	5.81
Cefixime	J01DD08	Oral	8	3.10
Cefixime and clavulanic acid	J01DD0	Oral	1	0.39
Cefpodoxime	J01DD13	Oral	2	0.78
Ceftriaxone	J01DD04	Parenteral	6	2.33
Cefuroxime	J01DC02	Oral	12	4.65
Ciprofloxacin	J01MA02	Oral	34	13.18
Clarithromycin	J01FA09	Oral	26	10.08
Clindamycin	J01FF01	Oral	2	0.78
Doxycycline	J01AA02	Oral	7	2.71
Erythromycin	J01FA01	Oral	1	0.39
Levofloxacin	J01MA12	Oral	17	6.59
Levofloxacin	J01MA12	Parenteral	2	0.78
Meropenem	J01DH02	Parenteral	1	0.39
Metronidazole	P01AB01	Oral	25	9.69
Nitrofurantoin	J01XE01	Oral	4	1.55
Ofloxacin	J01MA01	Oral	2	0.78
Ofloxacin	J01MA01	Parenteral	1	0.39
Secnidazole	P01AB07	Oral	9	3.49
Sparfloxacin	J01MA09	Oral	1	0.39
Trimethoprim/sulfamethoxazole	J01EE01	Oral	1	0.39

### Distribution of inpatient antibiotic use

Our results revealed that the majority of the patients (*n* = 27) received metronidazole in the course of their stay, followed by ceftriaxone (*n* = 24); 24 patients received levofloxacin infusion with 22 patients discharged with oral levofloxacin as their take-home drugs. In addition, amoxicillin and the clavulanic acid combination were received by 22 patients, with 19 patients taking oral amoxicillin and clavulanic acid combination as their take-home drugs (Table 5).

**Table 5.** Distribution of inpatient antibiotic use

Antibiotics	ATC	Route	*n*	%
Amikacin	J01GB01	Parenteral	1	0.53
Amoxicillin	J01CA04	Oral	1	0.53
Amoxicillin and clavulanic acid	J01CR02	Oral	19	10.16
Amoxicillin and clavulanic acid	J01CR02	Parenteral	22	11.76
Azithromycin	J01FA10	Oral	9	4.81
Cefixime	J01DD08	Oral	1	0.53
Cefotaxime	J01DD01	Parenteral	1	0.53
Cefpodoxime	J01DD13	Oral	3	1.60
Ceftazidime	J01DD02	Parenteral	1	0.53
Ceftriaxone	J01DD04	Parenteral	24	12.83
Cefuroxime	J01DC02	Oral	4	2.14
Ciprofloxacin	J01MA02	Oral	2	1.07
Ciprofloxacin	J01MA02	Parenteral	3	1.60
Clarithromycin	J01FA09	Oral	1	0.53
Clindamycin	J01FF01	Oral	1	0.53
Clindamycin	J01FF01	Parenteral	1	0.53
Levofloxacin	J01MA12	Oral	22	11.76
Levofloxacin	J01MA12	Parenteral	23	12.30
Meropenem	J01DH02	Parenteral	7	3.74
Metronidazole	P01AB01	Oral	9	4.81
Metronidazole	P01AB01	Parenteral	27	14.44
Moxifloxacin	J01MA14	Oral	1	0.53
Penicillin V	J01CE10	Oral	1	0.53
Piperacillin/tazobactam	J01CR05	Parenteral	1	0.53
Polymyxin B	J01XB02	Parenteral	1	0.53
Trimethoprim/sulfamethoxazole	J01EE01	Oral	1	0.53

### Distribution of antibiotic class and respective DDD

Fluoroquinolones belonging to the ATC code, J01MA, were the most prescribed antibiotics in both hospitalized patients (*n* = 51) and outpatients (*n* = 57). Penicillin and β-lactamase inhibitor belonging to the ATC code, J01CR, was next in both study groups with (*n* = 42) and (*n* = 52) for hospitalized patients and patients visiting the outpatient department, respectively. This was followed by the third-generation cephalosporins (*n* = 30) with the ATC code J01DD for hospitalized patients and macrolides (*n* = 41) for the general outpatient department (Table 6).

**Table 6.** Distribution of antibiotic class and respective DDD

Class	Code	Outpatient		Inpatient	
		DDD	*n* (%)	DDD	*n* (%)
Tetracyclines	J01A	1.71	7 (2.71)	—	0 (0.00)
β-Lactam/penicillin	J01CA	1.29	28 (10.85)	13.3	1 (0.53)
β-Lactamase sensitive penicillin	J01CE	—	0 (0.00)	3.8	1 (0.53)
Penicillin + β-lactamase inhibitor	J01CR	1.01	52 (20.16)	11	42 (22.46)
Cephalosporin (second generation)	J01DC	1.83	12 (4.65)	17.5	4 (2.14)
Cephalosporin (third generation)	J01DD	0.76	18 (6.98)	7.7	30 (16.04)
Carbapenems	J01DH	1	1 (0.39)	9.0	7 (3.74)
Sulphonamide and trimethoprim	J01EE	1	1 (0.39)	10	1 (0.53)
Macrolides	J01FA	2.33	41 (15.89)	17	10 (5.35)
Lincosamides	J01FF	0.38	2 (0.78)	9.4	2 (1.07)
Aminoglycosides	J01GB	1	1 (0.38)	5.0	1 (0.53)
Fluoroquinolones	J01MA	1.03	57 (22.09)	10.6	51 (27.27)
Polymyxin B	J01XB	—	0 (0.00)	4.0	1 (0.53)
Imidazole	J01XD	—	0 (0.00)	9.1	27 (14.44)
Nitrofuran	J01XE	0.75	4 (1.55)	—	0 (0.00)
Nitroimidazole	P01AB	0.65	34 (13.18)	5.6	9 (4.81)

### Outpatient antibiotic consumption rate

**Figure 1.**
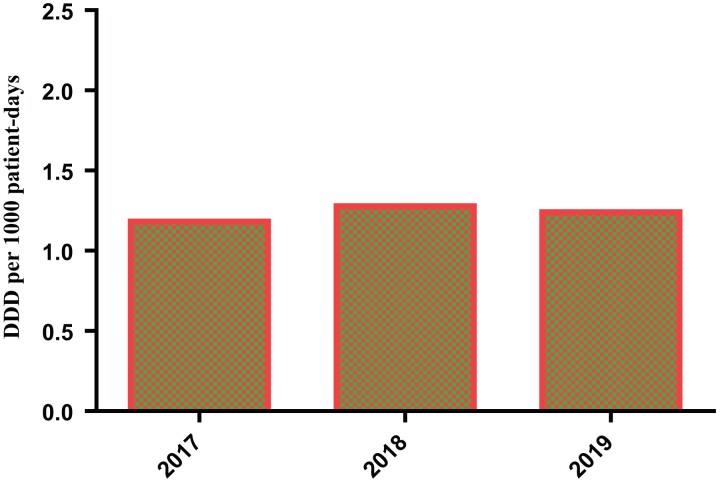
Comparison of the outpatient antibiotic consumption rate in terms of mean DDD per 1000 patient-days over 3 years.

The outpatient consumption rate is given as DDD per 1000 inhabitant-days. This provides a rough estimate of the proportion of the study population treated daily with a particular drug or group of drugs. From the study, the total DDD per 1000 inhabitants per day is 72.3 DDD/1000 inhabitant-days, and it can be inferred that the highest proportion of the study population in 2018 received the most daily use of antibiotics (Figure 1).

### Inpatient antibiotic consumption rate

**Figure 2.**
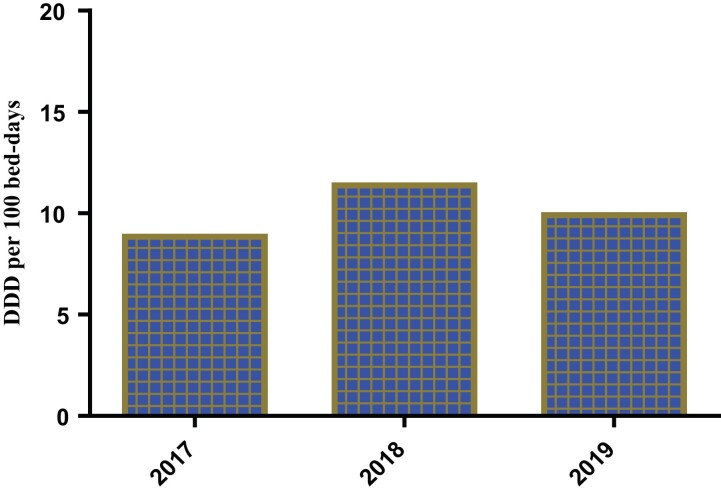
Comparison of the inpatient antibiotic consumption rate in terms of mean DDD per 100 bed-days over 3 years.

The antibiotic consumption rate for hospitalized patients is in DDD per 100 bed-days, with patients receiving the highest amount of antibiotics in 2018. Therefore, the total DDD/100 bed-days is 260.9 DDD/100 bed-days (Figure 2).

### Comparison of antibiotic consumption rate between inpatients and outpatients

**Figure 3.**
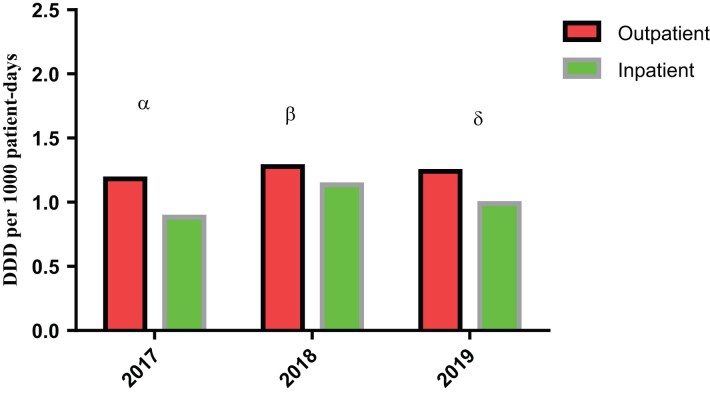
Comparison of antibiotic consumption rate between inpatient and outpatient. α, *P* = 0.019; β, *P* = 0.195; δ, *P* = 0.001.

Comparison of antibiotic consumption rate between the two study groups is given as DDD per 1000 patient-days with 2018 being the year of the highest DDD for both hospitalized patients and patients visiting the general outpatient department. The *P* values are 0.019, 0.195 and 0.001 for 2017, 2018 and 2019, respectively (Figure 3).

### Distribution of route of antibiotic administration

**Figure 4.**
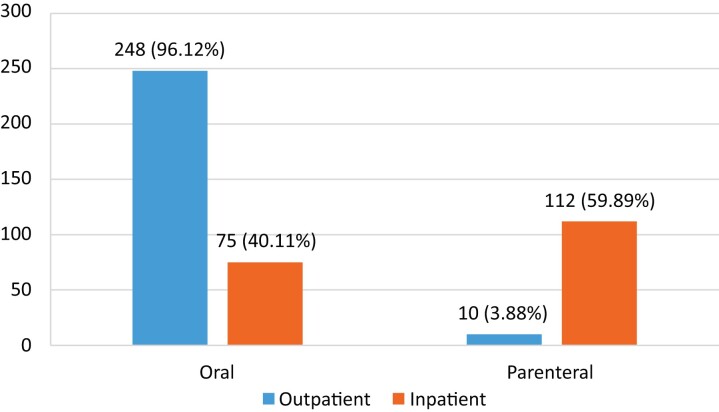
Comparison of distribution of route of antibiotic administration between inpatients and outpatients.

As expected, our findings revealed hospitalized patients received most of the parenteral medications, with about 59.9% of patients receiving IV fluids. However, only 40.11% of the hospitalized patients were discharged on oral medications. In contrast, 96.12% of patients visiting the outpatient department received oral medications, with 3.88% of the population receiving parenteral medications either in the form of IV or intramuscular (Figure 4).

## Discussion

The mean age of the population under study for people visiting the outpatient department was 43 years, while the mean age for hospitalized patients was 45 years.

One unique finding of our study revealed that females visited the hospital more than males. It is likely due to the economic implications of ill health being greater in females than males. The average number of antibiotics per prescription for people who visited the outpatient department was in the IQR of 1–2, while for hospitalized patients was in the range of 2–4, while the highest prescription had six different antibiotics (Table 1).

Peptic ulcer disease was the most frequent cause of hospital visits for patients who visited the outpatient department (Table 2). Clarithromycin (*n* = 26) was prescribed mainly by the general outpatient department as part of combination therapy for gastritis and peptic ulcer disease. This treatment regimen is in line with Nigeria’s national standard treatment guidelines. According to Egwuenu *et al*.,^9^ there is a high prevalence of peptic ulcer disease in Nigeria, and this is due to lifestyle habits such as smoking and excessive alcohol intake, which contributes to the corrosion of the gut lining.

Our findings also detected irrational prescribing of antibiotics in hospitalized patients. Instances such as using antibiotics for patients admitted for chronic kidney diseases, bilateral ventricular failure, nephrotic syndromes and malaria all indicate irrationality (Table 3). The national standard treatment guidelines do not at any point list the use of antibiotics for any of these disease conditions. However, antibiotics can sometimes be used to treat urinary tract infections in people with chronic kidney diseases observed in a study by Aloy *et al*. in 2020.^10^ Therefore, these groups need to make adequate dose and dosing regimen adjustments. The doses must be modified such that adequate therapeutic concentrations reach the kidney and, subsequently, the urine.^11^ Failure of these enhances the possibility of resistance emerging to the antibiotics used. Therefore, therapeutic drug monitoring is crucial for these groups.

Patients with nephrotic syndrome are at high risk of infection such as sepsis caused by *Streptococcus pneumoniae,* increasing their risk of mortality and morbidity. This is due to the loss of immune proteins, including immunoglobulin G (IgG) and alternative pathway complement components.^12^ This may be a suitable explanation of why antibiotics were used for these patients observed in this study. Antibiotics can be used to treat malaria, specifically co-trimoxazoles, macrolides, tetracyclines, fluoroquinolones and nalidixic acid derivatives. However, their uses are limited to severe and complicated malaria or in special groups with uncomplicated malaria.^13^ The class of antibiotics used were the third-generation cephalosporins, and there is no indication for their use clinically for this purpose.

A look at the indications for antibiotic use for outpatients also revealed several cases of irrational prescribing, although not as prominent as for hospitalized patients.

Amoxicillin and clavulanic acid combination drugs were the most prescribed by the outpatient department. This is due to the ability of the antibiotic to treat a variety of infections. WHO has classified it a critically important antimicrobial for human use.^5^ WHO went further to classify the antibiotics as prioritization factors 2 and 3. Prioritization factor 2 (P2) due to its high frequency of use of the antimicrobial class for any indication in human medicine or certain high-risk groups, since users may favour the selection of resistance and prioritization factor 3 (P3) due to the antimicrobial being used to treat infections in people for which there is already extensive evidence of transmission of resistant bacteria from non-human sources. WHO has also classified the antibiotic using the AWaRe classification under the Access group.^14^

Bacteria, viruses or fungi can cause nosocomial infections. For example, *Clostridioides* (*Clostridium) difficile*, a bacteria transmitted from an infected patient to others through healthcare staff via improperly cleansed hands, is one of the causes of these infections.^15^

Metronidazole, also classified by WHO into the Access group,^14^ is used to treat anaerobic infections, including those caused by *Bacteroides* and *Clostridium*.^16^ Metronidazole is also an antiprotozoal agent, and it is active against several protozoa, including *Giardia, Entamoeba histolytica* and *Trichomonas vaginalis*.^16^ Fluoroquinolones possess a broad spectrum of activity against many organisms, including aerobic Gram-negatives, such as Enterobacteriaceae, *Haemophilus* spp., *Neisseria* spp. and *Moraxella catarrhalis*.^17^ The effectiveness of metronidazole and levofloxacin against these organisms made them the right drug of choice for treating nosocomial infections, as observed in our study. However, levofloxacin has been classified by WHO under the Watch group, which means although it is included in the essential drugs list, it should be reserved for strictly MDR organisms.^14^

Our result compared with studies by Abubakar, 2020^18^ and Iliyasu *et al*., 2015^19^ on inpatient antibiotics prescription in Nigeria shows that less than 60% of patients received antibiotics for a bacterial infection. The most frequently prescribed antibiotic drug classes, as observed in their studies, were imidazole derivatives, cephalosporins, fluoroquinolones and β-lactams.^18^ The results of this study were no exception. This study also observed low generic prescription of antibiotics and high use of parenteral formulations as with previous studies. A finding supported by Iliyasu *et al*. in 2015.^19^

Generic prescription is necessary to reduce out-of-pocket expenditure, especially in Nigeria, where the patients bear the cost of medication. The use of parenteral formulations is costly and increases the risk of infection.^20^ Therefore, it should be reduced where possible.

Most antibiotics prescribed for both study groups were in brand names and not generic names; this finding agrees with similar studies.^19^ Lack of prescribers’ trust in generic substitutes and presumed therapeutic failure has been shown to influence generic prescribing.^21^

Antimicrobial resistance is a universal problem and a disaster waiting to happen; it is necessary to prevent antimicrobial resistance by methods such as rational prescription through institutionalized prescription audits or drug utilization studies.^22^ Sadly, many developing countries, including Nigeria, are yet to implement such measures, hence a rise in bacterial resistance.

The flow of antimicrobial resistance from hospitals into communities and vice versa makes it challenging to differentiate between community-acquired MDR infectious organisms and hospital-acquired ones.^23^

The total antibiotic consumption for hospitalized patients in this study was 260.9 DDD/100 bed-days (Table 6). In comparison with a study carried out by Hopkins in 2014 from New Zealand in which the antibiotic consumption for a secondary level facility was reported to be 117.6 DDD/100 bed-days,^24^ the DDD/100 bed-days for this study was higher. Our findings are slightly similar to studies by Sözen *et al*. in Turkey^25^ and Amaha *et al*. in Asmara, Eritrea.^26^ However, it was inconsistent with a study by Gutema *et al*.,^10^ who conducted a study in three medical wards of one of the largest tertiary hospitals in Ethiopia and reported that their antibiotic consumption was 91.8 DDD/100 bed-days. The higher figure obtained in this study reflects the large number of antibiotics that are being used in our hospitals.

Nigeria’s large population can also be a factor that was responsible for the sizeable antibiotic consumption figures, as Lagos University Teaching Hospital serves the large cosmopolitan city of Lagos state, Nigeria. DDD methodology does not also consider the reduction of doses in renal and hepatic failure patients, and in such cases, DDD would underestimate the number of antibiotics consumed. Antibiotic policies or prescribers’ specialty level might be some of the additional factors that could account for the difference between the hospitals.

The ATCC/DDD metric is beneficial in monitoring trends in drug use. For antibiotics, this could be important in improving antibiotic stewardship. Changes in the volume of DDDs, mainly where significant changes were observed, serve as a red flag warranting further studies to improve antibiotic use. In this study, the DDD of macrolides was highest (2.33), followed by second-generation cephalosporins (1.83) for patients visiting the outpatient department, while for hospitalized patients, second-generation cephalosporins were highest (17.5), followed closely by macrolides.^15^ Macrolides, second-generation cephalosporins, and fluoroquinolones are classified under the Watch group by WHO^14^ and as such they should be used carefully due to rapid development of resistance, but they still form one of the highest group used as observed in this study. These are some of the major contributors to the development of resistance. These antibiotics are prioritized for monitoring and they should be major targets for stewardship programmes.

Most prescribers understand that overprescribing antibiotics may lead to antibiotic resistance; however, they admit to the overuse of antibiotics and the prescription of antibiotics in the absence of bacterial infection.^27^ A multidisciplinary approach can improve the quality of antibiotic prescription, reducing cost and curbing infection/resistance.^28^ Interventions such as setting up antimicrobial stewardship committees; continuing in-service face-to-face medical education as a licensure requirement; and supervision, audit, and feedback systems effectively promote the rational use of antibiotics.^20^

According to our study, the antibiotic consumption rate for people visiting the outpatient department was highest in 2018 (Figure 1) using DDD per 1000 patient days, with 1.5 DDD per 1000 patient-days, while antibiotic consumption rate for hospitalized patients was also highest in 2018 (Figure 2) using DDD per 100 bed-days, with 15 DDD per 100 bed-days. This further emphasizes the high consumption of antibiotics by hospitalized patients.

The *P* values for antibiotic utilization for the 3 years indicated statistically significant differences between the antibiotic utilization in the years 2017 and 2019, but that there is no statistically significant difference in the antibiotic utilization for the year 2018 (Figure 3).

### Conclusions

We conclude that indiscriminate use of antibiotics is still being observed to be ongoing in the environment; not only are antibiotics misused, abused and overused, caution is not being used in the prescribing and use of these molecules. Instead, more antimicrobial molecules are being combined to tackle resistance without ensuring proper utilization of various programmes that help curb the emergence of resistance, such as antimicrobial stewardship programmes and the use of the national standard treatment guidelines.

A possible recommendation for good antibiotic stewardship includes the adoption of a pre-prescription approach, that is, antibiotics should not be dispensed without a prescription and should not be used in the hospital without informing the antibiotics steward or the infection specialist.

## References

[1] Raymond DP , Pelletier SJ, Sawyer RG. Antibiotic utilization strategies to limit antimicrobial resistance. Semin Respir Crit Care Med 2002; 23: 497–502. 10.1055/s-2002-35721 16088644

[2] Ayukekbong JA , Ntemgwa M, Atabe AN. The threat of antimicrobial resistance in developing countries: causes and control strategies. Antimicrob Resist Infect Control 2017; 6: 47. 10.1186/s13756-017-0208-x 28515903 PMC5433038

[3] Ichoku HE , Fonta WM. Catastrophic healthcare financing and poverty: empirical evidence from Nigeria. J Soc Econ Dev 2009; 11: 1.

[4] Godman B , Fadare J, Kibuule D et al Initiatives across countries to reduce antibiotic utilisation and resistance patterns: impact and implications. In: Arora G, Sajid A, Kalia V, eds. Drug Resistance in Bacteria, Fungi, Malaria, and Cancer. Springer, 2017; 539–576.

[5] WHO . Antimicrobial Resistance: Global Report on Surveillance, 2014 Summary. https://apps.who.int/iris/bitstream/handle/10665/112647/WHO_HSE_PED_AIP_2014.2_eng.pdf.

[6] Oduyebo OO , Olayinka AT, Iregbu KC et al A point prevalence survey of antimicrobial prescribing in four Nigerian tertiary hospitals. Ann Trop Pathol 2017; 8: 42. 10.4103/atp.atp_38_17

[7] Katakam P , Elfituri AA, Ramadan ZH et al A retrospective study on antibiotic use in different clinical departments of a teaching hospital in Zawiya. Libya. J Med Biomed Sci 2012; 4: 13–9.

[8] Maina M , Mwaniki P, Odira E et al Antibiotic use in Kenyan public hospitals: prevalence, appropriateness and link to guideline availability. Int J Infect Dis 2020; 99: 10–8. 10.1016/j.ijid.2020.07.084 32781162 PMC7562818

[9] Egwuenu A , Obasanya J, Okeke I et al Antimicrobial use and resistance in Nigeria: situation analysis and recommendations, 2017. Nigeria CDC/Nigeria Field Epidemiology and Laboratory Training Programme Second Annual Scientific Conference, Abuja, 2017. Abstract 2018:8(2).21.

[10] Gutema G , Håkonsen H, Engidawork E et al Multiple challenges of antibiotic use in a large hospital in Ethiopia–a ward-specific study showed high rates of hospital-acquired infections and ineffective prophylaxis. BMC Health Serv Res 2018; 18: 326. 10.1186/s12913-018-3107-9 29724214 PMC5934805

[11] Aloy B , Launay-Vacher V, Bleibtreu A et al Antibiotics and chronic kidney disease: dose adjustment update for infectious disease clinical practice. Med Mal Infect 2020; 50: 323–31. 10.1016/j.medmal.2019.06.010 31326299

[12] Mahalingasivam V , Booth J, Sheaff M et al Nephrotic syndrome in adults. Acute Med 2018; 17: 36–43. 29589604

[13] Gaillard T , Madamet M, Tsombeng FF et al Antibiotics in malaria therapy: antibiotics except tetracyclines and macrolides may be used against malaria. Malar J 2016; 15: 556. 10.1186/s12936-016-1613-y 27846898 PMC5109779

[14] WHO AWaRe Classification . https://www.who.int/publications/i/item/2021-aware-classification.

[15] Jayanthi A . Most Common Healthcare-Associated Infections: 25 Bacteria, Viruses Causing HAIs. Becker’s Hospital Review, 2014.

[16] Daley MJ , Hodge EK, Rose DT et al Antibiotic and antifungal therapy in the ICU. In: Salim A Brown C and Inaba K (eds.), Surgical Critical Care Therapy: Springer, 2018, 373–389.

[17] Moffa M , Brook I. Tetracyclines, glycylcyclines, and chloramphenicol. In: Bennett JE Dolin R and Blaser MJ (eds.), Mandell, Douglas, and Bennett’s Principles and Practice of Infectious Diseases: Elsevier, 2015, 322–8.

[18] Abubakar U . Antibiotic use among hospitalized patients in northern Nigeria: a multicenter point-prevalence survey. BMC Infect Dis 2020; 20: 86. 10.1186/s12879-020-4815-4 32000722 PMC6990515

[19] Iliyasu G , Dayyab FM, Bolaji TA et al Pattern of antibiotic prescription and resistance profile of common bacterial isolates in the internal medicine wards of a tertiary referral centre in Nigeria. J Glob Antimicrob Resist 2015; 3: 91–4. 10.1016/j.jgar.2015.02.005 27873676

[20] WHO . WHO Policy Perspectives on Medicines: Promoting Rational Use of Medicines. 2002. https://apps.who.int/iris/bitstream/handle/10665/67438/WHO_EDM_2002.3.pdf.

[21] Fadare JO , Ogunleye O, Iliyasu G et al Status of antimicrobial stewardship programmes in Nigerian tertiary healthcare facilities: findings and implications. J Glob Antimicrob Resist 2019; 17: 132–6. 10.1016/j.jgar.2018.11.025 30557686

[22] O’Neill J . Antimicrobial Resistance: Tackling a Crisis for the Health and Wealth of Nations. Review on Antimicrobial Resistance, 2014. https://amr-review.org/sites/default/files/AMR%20Review%20Paper%20-%20Tackling%20a%20crisis%20for%20the%20health%20and%20wealth%20of%20nations_1.pdf.

[23] Revelas A . Healthcare-associated infections: a public health problem. Niger Med J 2012; 53: 59–64. 10.4103/0300-1652.103543 23271847 PMC3530249

[24] Hopkins CJ . Inpatient antibiotic consumption in a regional secondary hospital in New Zealand. Intern Med J 2014; 44: 185–90. 10.1111/imj.12345 24528814

[25] Sözen H , Gönen I, Sözen A et al Application of ATC/DDD methodology to evaluate antibiotic use in a general hospital in Turkey. Ann Clin Microbiol Antimicrob 2013; 12: 23. 10.1186/1476-0711-12-23 24004538 PMC3847134

[26] Amaha ND , Weldemariam DG, Berhe YH. Antibiotic consumption study in two hospitals in Asmara from 2014 to 2018 using WHO’s defined daily dose (DDD) methodology. PLoS One 2020; 15: e0233275. 10.1371/journal.pone.0233275 32614832 PMC7332034

[27] Remesh A , Gayathri AM, Singh R et al The knowledge, attitude and the perception of prescribers on the rational use of antibiotics and the need for an antibiotic policy–a cross sectional survey in a tertiary care hospital. J Clin Diagn Res 2013; 7: 675–9. 10.7860/jcdr/2013/5413.2879 23730644 PMC3644442

[28] Adedapo AD , Akunne OO. Patterns of antimicrobials prescribed to patients admitted to a tertiary care hospital: a prescription quality audit. Cureus 2021; 13: e15896. 10.7759/cureus.15896 34322343 PMC8309689

